# Fertility, time to pregnancy, and pregnancy outcomes among women with recurrent miscarriages in the UK: a prospective observational longitudinal study

**DOI:** 10.1016/j.lanepe.2025.101343

**Published:** 2025-06-26

**Authors:** Constandina Koki, Rebecca Shields, Rebecca Sweetman, James Boyle, Omar Khan, Sarah N. Lim Choi Keung, Theodoros N. Arvanitis, Adam J. Devall, Nigel John Burroughs, Siobhan Quenby

**Affiliations:** a Warwick Mathematics Institute, University of Warwick, Coventry, CV4 7AL, UK; b Obstetrics and Gynaecology Department, Warwick Hospital, Warwick, CV34 5BW, UK; c Tommy's Charity, London, EC4R 0BB, UK; d Mathematics Institute, University of Oxford, OX2 6GG, UK; e Technology Strategy, Architecture and Standards Transformation Directorate, NHS England, Leeds, LS1 4AP, UK; f Networks Department, Unit ICT, Strategy & Policy, TNO Netherlands Organisation for Applied Scientific Research, The Hague, the Netherlands; g Department of Electronic, Electrical and Systems Engineering, University of Birmingham, Birmingham, B15 2TT, UK; h University Hospitals Coventry and Warwickshire NHS Trust, Coventry, CV2 2DX, UK; i Tommy's National Centre for Miscarriage Research, School of Medical Sciences, Department of Metabolism and Systems Science, University of Birmingham, UK; j Division of Biomedical Sciences, Warwick Medical School, University of Warwick, Coventry, CV4 7AL, UK

**Keywords:** Recurrent miscarriage, Subfertility, Infertility, Predictions, Risk factors, Pregnancy timeline, Miscarriage support

## Abstract

**Background:**

Recurrent miscarriage is a debilitating disorder associated with considerable physical and psychological morbidity. An estimated 50% of first trimester miscarriages remain unexplained. The aim of this study was to provide a personalised framework to guide the expectations of women experiencing recurrent miscarriage, with the ultimate goal of transforming clinical practice.

**Methods:**

We used real-world data from a UK longitudinal study of 1201 couples attending National Health Service (NHS) miscarriage clinics, with a history of previous miscarriages, comprising medical and obstetric history, results of investigations and pregnancy and neonatal outcome. We developed, parametrised, and validated predictive models for the probability that the next pregnancy is viable and for the time to next pregnancy. Time to next pregnancy separates couples into two groups, a group with subfertility, *i.e.,* delay in conception, and a group with no significant delay in conception. We used Bayesian inference for the latter model.

Trial registration number: The prospective data collections were pre-registered ISRCTN17732518; https://doi.org/10.1186/ISRCTN17732518.

**Findings:**

Predictive models of the time to pregnancy, the probability of the couple being subfertility and the probability of having a viable pregnancy can be parametrised from longitudinal study data. We identified several predictors for such models. In the viable pregnancy model, increased maternal age, higher Body Mass Index (BMI), having Polycystic Ovaries Syndrome (PCOS) and the number of previous miscarriages were associated with reduced odds of viable pregnancy. In contrast, having had previous live births increased the odds of a viable pregnancy. Model validation against a second external dataset gave an Area Under Curve (AUC) of 0.65 (95% Confidence Interval (CI): 0.55, 0.76). Of the 942 women referred to our recurrent miscarriage clinics and followed up over a period of 3 years, 10.7% (101) did not conceive during this time, indicating a potential subfertility problem. In the time to pregnancy model, increased maternal age, higher BMI, and smoking were associated with reduced likelihood of conception. Conversely, taking folic acid supplements and having a history of previous conceptions were associated with increased fertility. In our cohort, 53.4% (577 out of 1080 women) reported a pregnancy within 12 months. Additionally, 22.8% (277 out of 996 women who were followed up over a 2-year period) experienced a first pregnancy event in the second year. The area under the curve (AUC) for predicting pregnancy within 12 months was 0.60 (95% CI: 0.50–0.70) in an external validation using a second dataset.

**Interpretation:**

The pregnancy journey can be predicted on a personalised basis by integrating the validated models. We provide a framework for evidence-based management of women with miscarriage, comprising informed decision-making, including optimal referral to fertility services, and a tailored insight into fertility outcomes, thereby guiding expectations and providing psychological support.

**Funding:**

10.13039/501100022291Tommy's National Centre for Miscarriage Research.

**Research in context**

**Evidence before this study**

Couples who suffer miscarriage experience significant anxiety and depression and may continue to miscarry or not conceive again. A literature review was undertaken by the European Society of Human Reproduction and Embryology Recurrent Pregnancy Loss guideline group, in 2017 and updated in 2022 using PUBMED/MEDLINE and the Cochrane library. This review identified three prognostic tools, designed to provide an estimate of subsequent live birth in couples with recurrent pregnancy loss. However, the existing pre-conceptual study was limited by undefined or significant loss during follow-up. The other tools used a retrospective analysis of nationally collected registry data in Scandinavia. Registry data has the advantage of large numbers of patients, but is constrained by the limited small set of co-variates and includes only those women who formally registered their pregnancy thereby excluding individuals who did not register their pregnancy for various reasons. Prospective longitudinal cohort studies overcome these issues, by including women prior to conception and by collecting a larger set of covariates. We aimed to use longitudinal data from a UK cohort, to build a future pregnancy prediction model to inform couples before pregnancy who had previously miscarried, of their personal chance of a future successful pregnancy.

**Added value of this study**

In this study, we developed and validated two complementary models that together provide a pre-conception framework for predicting the reproductive trajectory following a miscarriage. Our models estimate the time to a future pregnancy and the likelihood of achieving a viable pregnancy across successive attempts and identify individuals at increased risk of subfertility, offering potential for earlier clinical recognition and support. To our knowledge, this is the first study to implement and combine these predictive approaches, and the first to integrate the resulting models into an online support tool for public use with evidenced-based advice. Our findings demonstrate the feasibility of generating individualised, data-driven risk predictions after miscarriage.

**Implications of all the available evidence**

We found that live birth after previous miscarriages can be predicted successfully pre-pregnancy, and this has been used in an online support tool accessed by more than 100,000 couples for advice and information about their future pregnancy prospects. A second model predicted which women were at risk of subfertility and estimated the time to a pregnancy event, which could be used for earlier referral for fertility investigations and treatment. Our models provide a framework for evidence-based management of women with miscarriage, comprising informed decision-making, including optimal referral to fertility services, and a tailored insight into fertility outcomes, thereby guiding expectations and providing psychological reassurance.

## Introduction

Miscarriage, the loss of a pregnancy prior to viability (24 weeks gestation), is common. An estimated 15% of pregnancies end in miscarriage: 10.8% of women experience one miscarriage, 1.9% experience two miscarriages, and 0.7% suffer three or more miscarriages.^1^ About 50% of first trimester miscarriages are due to foetal chromosomal anomalies,^2^ the remainder are often without explanation. The incidence of euploidic foetal loss increases with each additional miscarriage.^3^

Recurrent miscarriage is defined as the loss of two or more pregnancies,^4^ although a number of more stringent definitions are also used.^1^^,^^5^ Although an estimated 50% of first trimester miscarriages are due to chromosomal anomalies in the foetus,^2^ the remainder are often without explanation.

Recurrent miscarriage is a debilitating disorder associated with considerable psychological morbidity^6^ and miscarriage care guidelines recognise the importance of providing good physical care and psychological support.^4^^,^^5^ Thus, being able to guide couples on what to expect may help ameliorate the emotional and psychological impact. Couples are currently not provided with personalised estimates of their future chance of live births and doing so would be transformative for couples. Such a predictive tool requires detailed longitudinal studies. Known associations for miscarriage include, maternal age (>35 years) and the number of previous miscarriage,^7^, ^8^, ^9^ and a range of other factors are also implicated, including demographic, lifestyle, environmental, clinical factors among others.^1^

Previous work used retrospective analysis of nationally collected registry data in Scandinavia which has the advantage of large numbers of patients. However, this registry data is limited to a small set of co-variates and inclusion only of those who were able to register their pregnancy.^9^^,^^10^ Prospective longitudinal cohort studies overcome these issues and a larger set of (tailored) pre-pregnancy covariates can be incorporated.^7^^,^^8^^,^^11^^,^^12^ However, the nature of these studies entails smaller cohorts and can be limited by undefined or significant loss to follow up.

There is no national collection of data of miscarriage in England and Wales, so we analysed a UK longitudinal study of couples attending miscarriage clinics within the Tommy's National Centre for Miscarriage Research, comprising couples referred through the NHS referral system, see Supplement. We developed predictive models for the pregnancy timeline (time to pregnancy) and pregnancy outcome, specifically the probability of a viable pregnancy (>24 weeks gestation). These models were validated internally, and on an independent external cohort. The models were then used for an online support tool aimed at informing and encouraging women experiencing miscarriage but not receiving care post miscarriage.

## Methods

### Study design and population

We use two datasets: the first dataset was based on data from recurrent miscarriage clinics in University Hospital Coventry and Warwickshire (UHCW) (*CV*) and the second dataset was based on data from clinics at Birmingham Women's Hospital Foundation Trust (BWH) and Imperial College Healthcare NHS Trust (Imperial) London (*BL*). The data was collected from May 2017 until November 2021. The prospective data collections were pre-registered ISRCTN17732518; https://doi.org/10.1186/ISRCTN17732518. Ethics: REC Ref: 17/WM/0050: 17/WM/208. A bespoke electronic data collection and follow up system was devised, with 90% of clinic attendees at UHCW consenting to data collection and 82% answered follow-up text, clinic visits and/or telephone calls with information about further conceptions or lack of them.^13^ Miscarriage care followed European Society of Human Reproduction and Embryology ESHRE guidelines.^4^

**Fig. 1:**
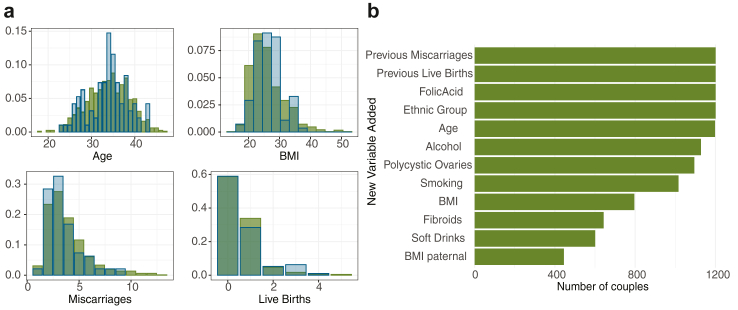
Summary of patient demographics for CV and BL datasets. (a) Distributions of age, Body Mass Index (BMI), previous miscarriages, and live births at entry to programme. Training dataset CV (green) and validation dataset BL (blue). Distributions are identical between cohorts (p > 0.05). (b) Covariate coverage with incremental variable addition, showing number of couples (pooled CV and BL datasets) who supplied that covariate and all those above it. Less than 50% of partners completed the clinic questionnaires so paternal covariates were not assessed.

The two datasets, initially comprised of 16 covariates, had similar demographics, Table 1, and covariate distributions of the two datasets were not statistically different (p-value = 0.01, Storer and Kim^14^), Fig. 1a.

**Table 1:** Demographic statistics and summary of pregnancy outcomes of the CV and BL cohort.

	Training data CV	Validation data BL
**Cohort characteristics**		
Number of couples	933	268
Average age (years)	33.90 (SD: 5.04, n = 933)	34.02 (SD: 4.91, n = 268)
Average previous miscarriages	3.43 (SD: 1.82, n = 933)	3.60 (SD: 1.64, n = 268)
Average previous live births	0.46 (SD: 0.75, n = 933)	0.44 (SD: 0.73, n = 268)
Average BMI	26.44 (SD: 5.67, n = 756)	26.91 (SD: 6.54, n = 147)
Polycystic ovaries syndrome diagnosis	144 (17%, n = 850)	55 (21%, n = 260)
Taking folic acid supplements	466 (50%, n = 933)	118 (44%, n = 268)
Smokers	65 (8%, n = 808)	30 (12%, n = 252)
Drinking alcohol	484 (56%, n = 865)	149 (56%, n = 266)
Ethnicity	White, Mixed, Black	White, Mixed, Black
**Summary outcomes**		
Total pregnancies	951	153
Viable pregnancies	579 (60.9%)	90 (58.8%)
Miscarriages	372 (39.1%)	63 (41.2%)

Brackets report the standard deviation and the sample size. Soft drink consumption and fibroid diagnosis were not recorded for BL, so these covariates are not shown. PCOS diagnosis was made by an NHS clinician prior to entering the study. In the combined CV + BL dataset 78% of women were White (937 out of 1201), 10% were of a mixed heritage (120 out of 1201), and the remaining 12% (144 out of 1201) were Black. Total pregnancies refer to all pregnancies recorded in the dataset, including multiple pregnancies per woman.

Missing values were completely at random (p = 0.073, non-parametric rank test^15^). Covariate coverage for the first 12 covariates (ranked by coverage/completeness) is shown in Fig. 1b. We restricted analysis to covariates with substantial coverage (thus excluding any beyond the 12 shown) and present in both datasets (CV and BL), which excludes paternal Body Mass Index (BMI), soft drinks and fibroids that were only in the CV dataset. Thus, 9 covariates were analysed. We used the backward selection model with a significance level of 5% to remove covariates not supported by the data; in particular Black or Asian ethnicity was not significant in any model, likely a consequence of the low ethnic diversity in the dataset. For each model, we kept the statistically significant covariates or the set of covariates that provided the best predictive performance. The pregnancy outcomes for the women in the study are summarised in Table 1. Note, that the miscarriage rate per pregnancy is about 40% in these cohorts (372 out of 951 in CV and 63 out of 153 in BL), substantially higher than the 15% in the general population.^1^ This emphasises that the women in the study were at a high risk of miscarriage.

### Statistical methods and analysis

We developed predictive models for the pregnancy timeline (time to pregnancy from first clinic visit) and pregnancy outcome, specifically the probability of a viable pregnancy (>24 weeks gestation). We separated the analysis into an analysis of pregnancies classified into viable pregnancy (pregnancy beyond 24 weeks, or birth) and miscarriage (pregnancies under 24 weeks are excluded), and also analysed dependencies for the time to pregnancy, including the women who report no pregnancy. Some patients may not experience a pregnancy event during their time in the program (ranging from 6 months to 4 years) but may still be able to conceive. Nevertheless, early identification of individuals with potential subfertility concerns is crucial to ensure timely referral and appropriate management. The integration of these models can provide a detailed, personalised framework, while also offering couples comprehensive information about their pregnancy journey.

A logistic regression model for viable pregnancy (pregnancies lasting >24 weeks) versus miscarriage was fitted to the CV dataset, details in Supplementary Text. The model was fitted to the training dataset (CV) on all pregnancy events (*i.e.,* multiple pregnancy events for a patient are incorporated, changing appropriately the pregnancy history and age, n = 784 couples) assuming each pregnancy is independent; a model fitted only on the first pregnancy event gave near identical results, Supplementary Table S1.

A proportional hazards model could be used to analyse time to pregnancy; however, this assumes all patients will fall pregnant eventually. Since we have evidence of (discrete) heterogeneity in our population, specifically a group with a subfertility problem that will not get pregnant, we used a cure rate model,^16^ and used joint prediction of whether a patient is in the fertile or subfertility problem group, and the time to pregnancy for the fertility group, see Supplementary Text. In this model, we used age over 35 years (at the entry to study), BMI above 25 kg/m^2^, taking folic acid supplements, smoking, previous conceptions as the sum of previous miscarriages and previous live births, as covariates. Because the effect of BMI is nonlinear, with both high and very low BMI being implicated in infertility,^17^ we used BMI over 25 kg/m^2^ as covariate, *i.e.,* for women with a BMI below 25, this covariate was set to 0.

We parametrised and validated the viable pregnancy and the subfertility problem-time to pregnancy models. We only retain statistically significant covariates; the training set can therefore increase since requiring additional covariates is more stringent, see Supplementary Text. Model performance was calibrated using the Receiver Operating Characteristic (ROC) analysis. We quote Area Under the Curve (AUC) which measures model performance across all choices of threshold (for instance on sensitivity or specificity). An AUC of 1 would be perfect prediction, an AUC of 0.5 would be purely random. We report results for self-validation (see also Supplementary Text) and external validation on the independent BL dataset.

Statistical analyses were performed in Rstudio (version 2025.05.0, “Mariposa Orchid”, running R version 4.5.0; R Core Team 2025) and bespoke Python (version 3.13.3) in Jupyter Notebook (version 7.0.8).

### Role of the funding source

The funding source supported the development of the technical and governance infrastructure to facilitate data access for research and the analysts time to conduct the study. The funders played no role in study design and protocol development, data curation and analysis, interpretation or writing the manuscript.

## Results

**Fig. 2:**
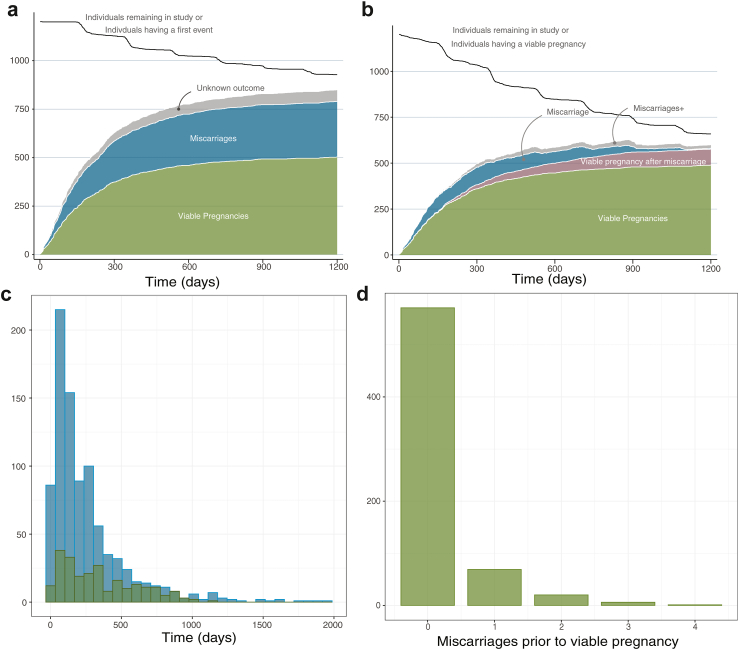
Outcome summary (pooled CV and BL datasets). (a) Stacked first event outcome with time on programme (counts). Cumulative events in both CV and BL datasets showing (stacked) viable pregnancy (>24 weeks) (green), a miscarriage (blue), and an ongoing or a reported pregnancy but without confirmed outcome (grey). (b) Decomposition of couples that have a viable pregnancy as first event on study (green) or after a miscarriage on study (pink). The number of couples with one miscarriage (blue) or multiple miscarriages (grey) on study before having a viable pregnancy is shown through by time. The total number of couples remaining in the study (including having a viable pregnancy) is shown (black line). (c) Histogram of time to first (blue) and second (green) event. (d) Histogram of the number of miscarriages before a viable pregnancy while on program. Outcome summary for CV dataset alone is shown in Supplementary Figure S2.

Cumulative outcomes–miscarriage and viable pregnancy—on both datasets, are shown in Fig. 2; cumulative outcomes only on CV dataset can be found in Supplementary Figure S2. Most, 80.9% of pregnancies occurred within the first year, whilst there remained a proportion of women who failed to report a pregnancy.

In particular, 19.3%, (192 out of 996) of couples that were followed up in a period of 2 years, and 10.7%, (101 out of 942) of couples that were followed up in a 3-years period, did not report an event, suggestive of a subfertility problem.^18^ 53.4% of women reported a pregnancy within 12 months (577 women out of n = 1080 couples in follow up), and 22.8% had a first event in the second year (277 out of n = 996 couples in follow up). There is thus heterogeneity in the study group with (at least) two groups: couples with subfertility problem (which could be due to various reasons including female or male infertility, psychological issues, e.g., fear of miscarriage),^13^ and a fertile group with a large variation in the time to achieve pregnancy. The time to conception curve within our recurrent miscarriage cohort is exponential with mean time 348 days, Supplementary Figure S1.

### Predicting a viable pregnancy/the risk of miscarriage per pregnancy

**Fig. 3:**
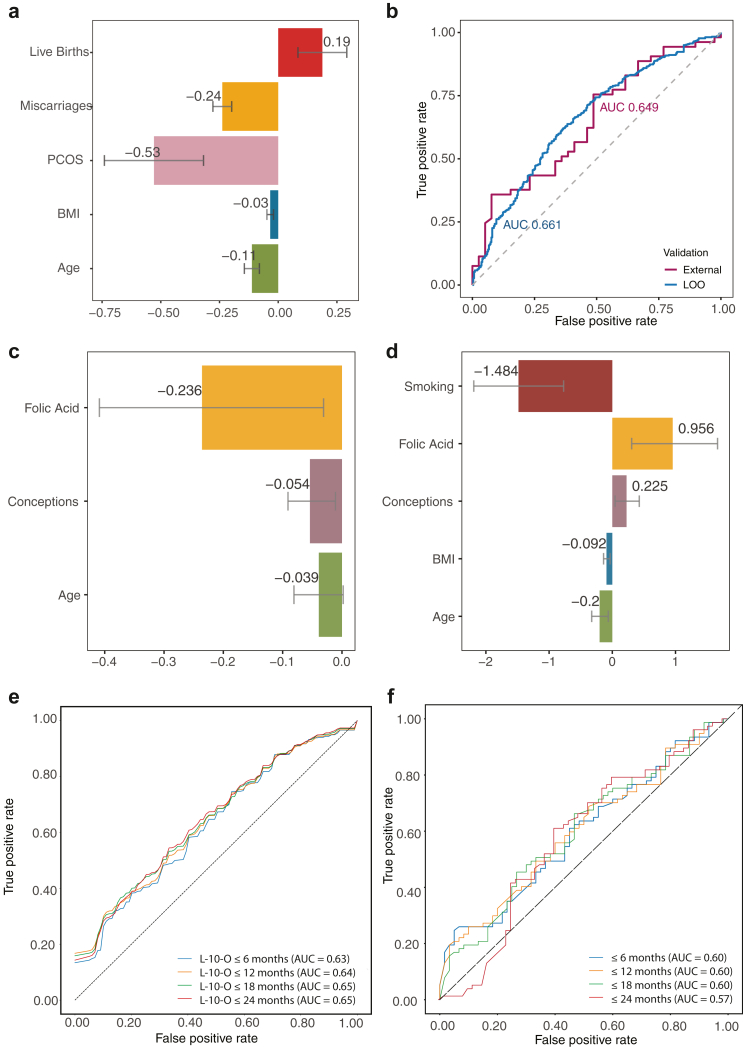
(a/b) Viable pregnancy model. (a) Significant covariates: estimates are consistent across the datasets. Each bar shows the estimate of significant covariate while the grey lines denote the 95% error bars. (b) Receiver operating characteristic (ROC) curve and Area Under Curve (AUC) for the viable pregnancy model, using the CV dataset (blue) and with external validation on BL dataset (red). (c-f) Time to pregnancy and fertility model trained on CV dataset. Posterior mean estimates for (c) Time to pregnancy covariate dependence, and (d) for fertile class covariate dependence. Bars denote the 90% highest density credible intervals. (e/f) ROC curves for achieving a pregnancy within the specified time interval. (e) Leave-k-out (LkO) validation, with k = 10. (f) External validation on BL dataset.

The significant covariates (5% significance level, using a backward selection method) are previous miscarriages, maternal age (at pregnancy), polycystic ovaries and BMI, Table 2 and Fig. 3a. Previous live births has borderline significance at 5.3% and is retained in the model. Previous live births reduces the risk of miscarriage consistent with previous reports,^9^; all other significant covariates increase the risk.

**Table 2:** Viable pregnancy model for analysing multiple pregnancies when trained on CV data set and validated with Leave-one-out (LOO) and externally on BL dataset.

Covariate	Mean (SE)	Odds ratio per unit (95% CI)	p-value
Intercept	2.45 (0.42)	11.61 (4.99, 25.81)	<0.0001
Age	−0.11 (0.03)	0.89 (0.84, 0.95)	<0.001
BMI	−0.03 (0.01)	0.97 (0.94, 0.99)	0.02
PCOS diagnosis	−0.53 (0.21)	0.59 (0.39, 0.89)	0.01
Previous miscarriages	−0.24 (0.04)	0.79 (0.73, 0.85)	<0.0001
Previous live births	0.19 (0.10)	1.21 (1.00, 1.49)	0.05

Area under curve (95% CI)	
LOO	0.66 (0.62, 0.70)
External BL, n = 92	0.65 (0.55, 0.76)

Calibration	
Calibration in the large	−0.28 (−0.71, 0.15)
Calibration slope	1.05 (0.17, 1.95)

Observed/expected ratio	
External BL	0.91 (0.75, 1.05)

We report the mean estimated parameters (with standard error), the odds ratio per unit (95% CI) and p-values for each covariate. Averaged predicted risk (calibration in the large), calibration intercept and observed versus expected (predicted) ratio, are reported for external validation (95% CI). Odds are adjusted for other significant covariates.

**Fig. 4:**
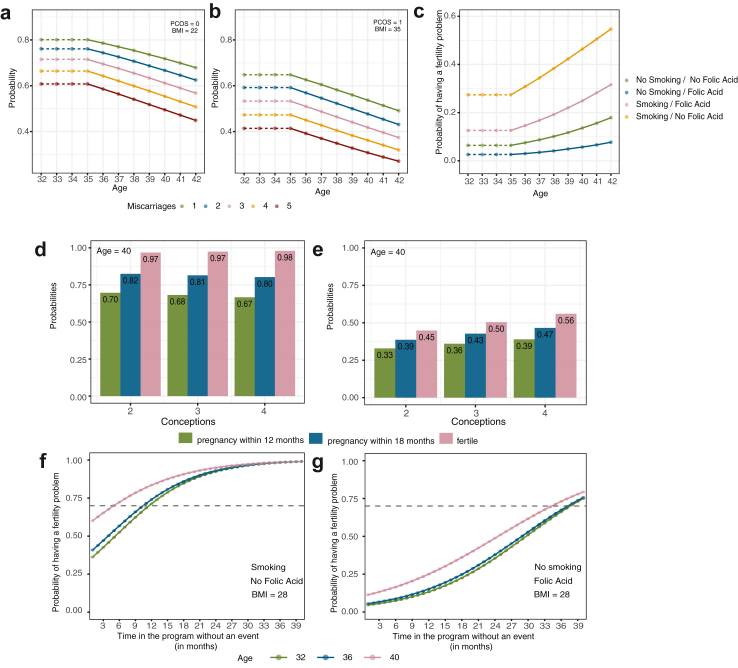
Example of a detailed evidence-based framework for determining a suitable referral time to fertility services. (a/b) Viable pregnancy predictions with age: Shown are predictions for women with the stated number of previous miscarriages and no previous live births. (a) Normal BMI, no PCOS. (b) Obese, PCOS. Probabilities are independent of taking Folic Acid or smoking. (c) Probability of having a subfertility problem by maternal age. Various cases shown smoking, non-smoking, and using folic acid supplements. Women with BMI < 25, 3 previous miscarriages, and no previous live births. Probabilities are independent of PCOS diagnosis. Recall age dependence is only modelled post 35: women 35 and under have the same age dependent risk. (d/e) The probability of having a pregnancy within 12 months (green), within 18 months (blue), and the probability of being in the fertility class at entry of the program (pink) for: (d) women at 35, normal BMI (<25), taking folic acid supplements, and not smoking and (e) women at 40, overweight (BMI = 27), not taking folic acid supplements, and smoking. Probabilities vary with the number of conceptions. (f/g) Assessment of risk of a couple having a subfertility problem given the duration on the program without a pregnancy. (f) Smoking and no folic acid and (g) not smoking and taking folic acid supplements. Women have a BMI = 28 (overweight), 2 previous conceptions prior to entering to the study, and of ages 32, 36, and 40 yrs as shown. Grey dashed line corresponds to a referral threshold of 70% for the estimated probability of having a subfertility problem.

Model validation gave an AUC of 66.1% under the internal Leave-One-Out (LOO) analysis, and an AUC of 64.8% on external validation against the BL dataset, Table 3 and Fig. 3b. We also carried out a calibration analysis,^19^ Table 2; there was no evidence of overestimation or overfitting, Supplementary Text. A model fit to the BL data set gave similar results, Supplementary Table S1, showing that the predictive model is robust across different datasets. Age (above 35 years) and previous miscarriages are predicted to cause a reduction in the probability of a viable pregnancy, with an odds ratio of 0.89/year and 0.79/miscarriage respectively, Table 2. A PCOS diagnosis gives a substantial reduction in the probability of a viable pregnancy, whilst increasing BMI progressively decreases the viable pregnancy probability with an odds ratio 0.97 per kg/m^2^. Fig. 4a and b presents examples of predicting a viable pregnancy for women with different age, BMI, and PCOS.

**Table 3:** Time to pregnancy and fertility model.

Covariate	Time to pregnancy		
	Mean (90% BCI)	Hazard ratio	Tail probabilities
Intercept	−5.30 (−5.49, −5.12)	–	0.00
Age	−0.04 (−0.08, −0.002)	0.96	0.05
Folic acid	−0.24 (−0.41, −0.03)	0.79	0.01
Previous conceptions	−0.05 (−0.09, −0.01)	0.95	0.01

Covariate	Fertility model		
	Mean (90% BCI)	Odds ratio	Tail probabilities
Intercept	2.01 (0.13, 2.46)	7.46	1.00
Age	−0.20 (−0.33, −0.06)	0.82	0.02
BMI	−0.09 (−0.14, −0.04)	0.91	<0.002
Folic acid	0.96 (0.31, 1.67)	2.60	0.01
Smoking	−1.48 (−2.19, −0.77)	0.23	<0.001
Previous conceptions	0.23 (0.05, 0.43)	1.25	0.02

	Area under curve (95% CI)	
	LkO	External BI
6 months	0.63 (0.57, 0.67)	0.60 (0.50, 0.69)
12 months	0.64 (0.59, 0.70)	0.60 (0.50, 0.70)
18 months	0.65 (0.59, 0.70)	0.60 (0.50, 0.70)
24 months	0.65 (0.58, 0.70)	0.57 (0.50, 0.67)

Calibration	
Calibration in the large (12 months)	−0.34 (−0.68, 0.00)
Calibration slope (12 months)	1.04 (0.00, 2.05)

Observed/expected ratio	
12 months	0.88 (0.76, 1.01)

We report the posterior mean estimations for each covariate (90% Bayesian Credible Interval) and the associated posterior tail probabilities, analogous to p-values. Model was trained on the CV dataset and validated on the BL dataset or through Leave-k-Out (LkO, k = 10) for achieving a pregnancy within a specified interval, and we report the Area Under Curve (95% CI). Averaged predicted risk (calibration in the large), calibration intercept and observed versus expected (predicted) ratio, are reported for predicting a pregnancy within 12 months using external validation. The time to pregnancy (of a couple in the fertile group) is e^−βX^ and the odds of being in the fertile group are e^αX^. For instance, the mean estimated time to pregnancy for a woman of age 36, who takes folic acid and has 4 previous conceptions is 215 days.

### Predicting a fertility problem and the time to pregnancy

Age (at entry to study) over 35, BMI (over 25 kg/m^2^), taking folic acid supplements, smoking, and previous conceptions were all significant covariates for prediction of a subfertility problem, with previous conceptions and folic acid supplements reducing the predicted risk of delay in conception, Table 3 and Fig. 3c and d.

For the fertile group, time to pregnancy increased with age, folic acid supplements and previous conceptions. For example, as shown in Table 3, a woman that is one year older than the another woman has 4% lower chance of conceiving at specific time t, while women who take folic acid supplements have 21% lower chance of conceiving at time t compared to women who do not take folic acid supplements. Model validation was based on the prediction for achieving a pregnancy within a specified interval, using LkO analysis (within the CV data set), AUC 0.63–0.65, and on external validation against the BL dataset, AUC 0.57–0.60, Table 3 and Fig. 3e and f.

The estimated probability of being in the fertility problem group by maternal age for various cases, indicating the effect if each covariate on the estimated probability, is plotted in Fig. 4c.

Fig. 4d and e, show how the estimated probabilities of having a pregnancy within 12 and 18 months and the probability of being in the fertility class vary for 35 and 40-year-old women when allowing for different BMI, smoking or not and taking or not taking folic acid supplements. In Fig. 4f and g, we plot 2 examples of the probability of being in the subfertility problem group based on the duration with no pregnancy. For instance, to achieve a predicted 70% chance of being in the subfertility problem group, a period of 3 months without a pregnancy is required for a 40-year-old woman who smokes and doesn't take folic acid supplements, Fig. 4f, whilst for a 40-year-old woman who takes folic acid and doesn't smoke, the duration is 33 months, Fig. 4g. Thus, a threshold on the probability of the couple being in the subfertility problem group can be used to set a referral time dependent on the couples' demographic data. The referral time for couples without an event under a 70% threshold criterion is shown in Supplementary Figure S6, against women's age and BMI. This model provides an evidence-based framework for determining a suitable referral time to fertility services.

### Validation on subgroup predictions

**Fig. 5:**
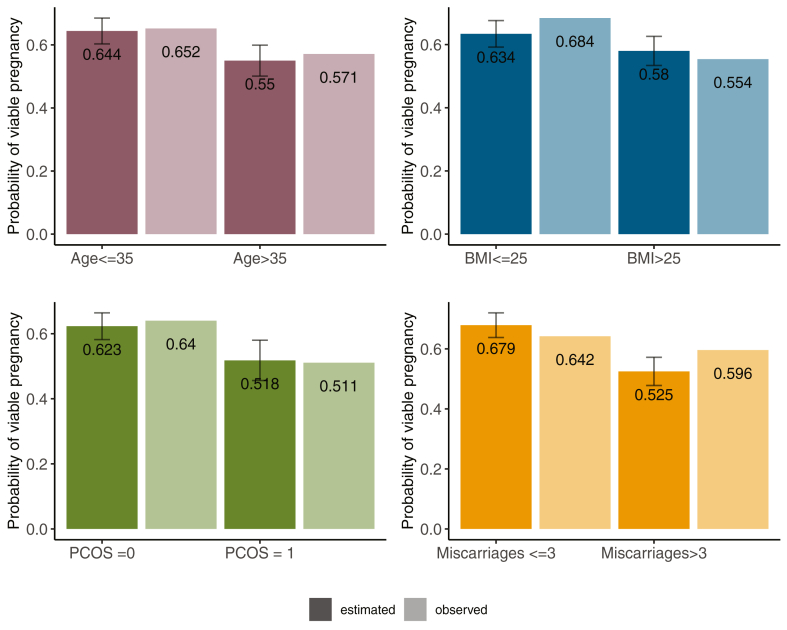
Assessing viable pregnancy model performance on patient subgroups. Trained on CV: RFE002 and validated on CV: RFE003 + BLI: RFE005. Dark columns show the averaged predicted probabilities of a viable pregnancy for each subgroup of BL dataset. Error bars denote the standard deviation of the predicted probabilities.

The effects of individual covariates are weak, with low AUC in both models, Fig. 3; therefore, only probabilistic individualised predictions can be given, and not certainty of an event/outcome. We can illustrate the accuracy on defined subgroups, using individualised predictions on each couple in the cohort. To achieve reasonable confidence, we split the CV cohort (splitting the two clinics) and trained the model on data on CV: RF002, and validated on the pooled data from CV: RF003 + BL. We partitioned the validation cohort into 2 subgroups of around 150 patients divided by one of the strongest covariates, for instance the 2 age groups–under and over 35 years. In each subgroup the predicted and observed probabilities of a viable pregnancy are consistent, Fig. 5. However, the differences in probability are small since the covariates are weak; for example, the probability of a viable pregnancy for women with PCOS versus without PCOS is reduced by 16.9%, while the probability of a viable pregnancy for women with a history at least 3 miscarriages versus women with less than 3 miscarriages is reduced by 22.7%, Fig. 5. Subgroups based on two covariates show larger differences, Supplementary Figure S4.

### Predicting the viable pregnancy journey

**Fig. 6:**
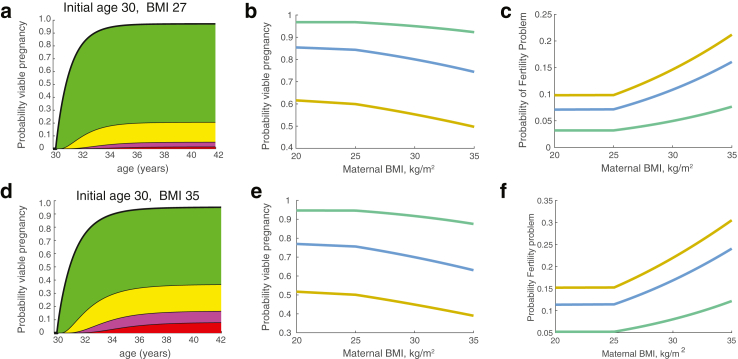
Combined model predictions. (a) Probability of a viable pregnancy (green), with 1, 2, 3 miscarriages then viable pregnancy (yellow, magenta, red respectively) with time. Example for a woman with initial age 30, 2 previous miscarriages, no live births, BMI 27 kg/m^2^, taking Folic Acid supplements, non-smoker, and no PCOS. Probabilities of 0, 1, 2, 3, miscarriages before viable pregnancy are 78%, 16%, 3.8%, 1.5% respectively. (b) Probability of having a viable pregnancy at age 42 with maternal BMI. (c) Probability of being in the subfertility problem group. Illustrated in (b), (c) for a woman aged 30 (green), 38 (blue), 40 (orange), 2 previous miscarriages, no live births, taking Folic Acid supplements, non-smoker, and no PCOS. (d–f) Women with 4 previous miscarriages and a history of PCOS. Illustrated for a woman with BMI 35 kg/m^2^. Probabilities of 0, 1, 2, 3, miscarriages before viable pregnancy are 53%, 21%, 9%, 8% respectively.

The models for a viable pregnancy and the time to pregnancy can be utilised together to construct a prediction for future pregnancy outcomes and timings, or the pregnancy journey, *i.e.,* modelling the time to a first pregnancy, through a possible second, third etc. until a viable pregnancy is achieved. It allows us to predict the probability of a viable pregnancy on the first, second or later pregnancy, and the time distribution to achieve a viable pregnancy, Fig. 6, Supplementary Diagram S1. The probability of having a viable pregnancy with time (age) is shown in Fig. 6a for women currently aged 30 years, 2 previous miscarriages and taking folic acid supplements. The cumulative probability of having a viable pregnancy rises to 0.96 after 12 years, with only a 22% probability of a miscarriage before a viable pregnancy. The probability of having a fertility problem by 40 is 4%. The predicted probability of having a viable pregnancy by age 42 decreases with BMI and current age, whilst the probability of having a fertility problem increases, Fig. 6b and c. A woman with higher risk factors is shown in Fig. 6d–f. The probabilities of a viable pregnancy or having a fertility problem by age 40 with age and BMI is shown in Supplementary Figure S6.

## Discussion

We report two predictive pre-conception models for natural conception parametrised from a longitudinal study of 1201 women referred to an NHS tertiary miscarriage clinic and validated externally on a further cohort of 288. The NHS referral system provides a unique opportunity to study natural conception within a real-world setting, since accessing assisted reproductive technology treatments is delayed through the NHS. To the best of our knowledge, there is no other national longitudinal collection of such data in England and Wales.

Different models were needed to predict conception and pregnancy viability, where viable pregnancy is defined as foetal heart beating after more than 24 weeks gestation (a surrogate for live birth). Prediction of future subfertility could allow interventions and early referral to fertility services at a younger age when treatments are more likely to be successful.

Our viable pregnancy model reproduces previous reports that age, BMI and previous miscarriages are significant risk factors for miscarriage,^1^ and a previous report that previous live births reduce this risk.^9^ Whilst the association between PCOS and miscarriage has been described previously,^20^ our detailed prospective data allowed us to incorporate this diagnosis into our model. The viable pregnancy prediction model, with covariates age, number of previous miscarriages, BMI, PCOS, and previous live birth was used in the Tommy's Miscarriage support tool, https://www.tommys.org/baby-loss-support/miscarriage-information-and-support.

Analysis of the time to pregnancy identified couples who had been referred to a recurrent miscarriage clinic who have a subfertility problem; 10.7% (101 out of 942) did not report a pregnancy event within 3 years in follow up.

Some factors had differing effects on the chance of a viable pregnancy and subfertility. An increasing number of previous miscarriages increased the miscarriage risk but reduced the probability of subfertility (the presented model uses the number of previous conceptions, that includes previous miscarriages; however a model separating previous miscarriages and live births showed both live births and previous miscarriages reduce subfertility). This suggests that repeated miscarriage and subfertility problems have differing aetiologies, and imply that miscarriages were not part a pathway of ovarian ageing, agreeing with the finding that anti mullerian hormone was not helpful in predicting live birth in recurrent miscarriage.^12^

Maternal age increased the time to pregnancy and the risk of miscarriage. In our cohort a 5.8% and 8.7% probability of a subfertility problem for age groups 27–32 years, and 33–38 years respectively was similar to the general population.^21^

Taking folic acid increases the probability of being fertile, as observed before,^22^ but delayed the time to pregnancy. This is because folic acid increases fertility, and women who would otherwise be unable to conceive likely take longer to conceive.

Our study has several limitations. Our externally validated predictive models are only applicable to women with recurrent miscarriage and similar demographics; however since the risk factors that we identified are common for all women the models may be applicable to a wider and more diverse population, but without a more general population dataset we cannot confirm or validate this. The covariates reported by couples attending the recurrent miscarriage clinics and the cohort heterogeneity limit the models general applicability, in particular we had insufficient ethnic diversity in the cohort to capture its effects,^1^ (ethnicity was not significant in our models). Finally, our models have limited predictive power, with AUC 57–65% on external validation. This prediction accuracy is typical of models of reproductive studies, where reported AUCs range from 60% to 70%.^9^^,^^23^, ^24^, ^25^ The predictive power is likely impacted by the sample size and patient heterogeneity, although it may reflect an inherently weak association between miscarriage and the available covariates.

The power to assess ethnicity was low in our data set since our cohort comprised predominantly Caucasian women, whilst it is known that recurrent miscarriage is more common in black women.^1^ The dependence on BMI should also be reassessed within the context of ethnicity, since the distribution and percentage of body fat varies with ethnicity, in particular Asian populations have lower thresholds for overweight (23 kg/m^2^) and obese (25 kg/m^2^), compared to White populations, 25 kg/m^2^ and 30 kg/m^2^ respectively. Further, since BMI is a proxy, it is unknown if body mass composition affects miscarriage and fertility.^26^

There are other factors that have been suggested to affect the risk of miscarriage that we were unable to assess. This includes uterine anomalies, the endometrium state,^27^ other clinical measurements, paternal factors, genetic risk factors,^28^ and diet,^29^ whilst the previous sequence of births/miscarriages and previous pregnancies' birth weight also affect outcome in next pregnancy.^9^ Such reports suggest outcome is affected by a large range of factors, each to a small degree, implying prediction requires extensive and updated measurements, although ruling out that these are confounders on fewer (possibly unknown) variables is currently not possible. The limited predictive power of our models (AUC 57–65%) is related to the fact that the covariates are weak. For instance, the predicted probability of a viable pregnancy rarely approaches 0 or 1 for any individual couple, thus random chance is never eliminated. This raises the fundamental question of whether more accurate predictions are possible, *i.e.,* whether the limit on prediction is determined by random effects (noise)–environmental, physiological, and psychological fluctuations–improvements in prediction are then not possible, or whether there are covariates that have not been determined to date or if covariate dependence is infact masked by miscarriage being an heterogeneous condition, and thus population stratification is required. A larger prospective study would allow more covariates to be assessed, population heterogeneity and miscarriage stratification performed, and the functional dependence on age and BMI (nonlinear) improved. The predictive power of the models would then likely improve.

Our study was based on pre-conception risk factors. Predicting outcome prior to pregnancy is needed for the implementation of preventative interventions. Pregnancy prediction is important because women with recurrent miscarriage are at increased risk of other adverse obstetric outcomes, including preterm birth, placental disorders,^1^ suggesting common causes.

Evidence based management of recurrent miscarriage is a difficult task since there are few absolutes and so advice to couples must incorporate discussion of the element of chance. The high use of the Tommy's Miscarriage Support Tool (more than 100,000 couples have already used it in 2 years), is a strong endorsement of probabilistic based advice. BMI, smoking, and diet are known factors that reduce the chances of a viable pregnancy through the combined factors of reduced fertility, longer periods to get pregnant and a higher risk of miscarriage. The time scale for changes in lifestyle to increase the chance of a viable pregnancy, for instance reducing maternal BMI, quitting smoking, and improving diet, is however unknown. Quantifying the time scale across lifestyle changes will be a challenge given the need to quantify these covariates, and the underlying randomness.

Future studies with more comprehensive datasets may benefit from incorporating a wider range of potentially informative variables. These include maternal factors such as diet, stress, uterine anomalies and endometrium factors, environmental factors such as pollution, and paternal factors such as age, BMI, lifestyle, and health history.^1^^,^^6^ Longitudinal reproductive histories, including sequence and type of pregnancy complications and live births; may also carry predictive value.^10^ The integration of such data may help to reduce unexplained variance and enable more refined, personalised risk predictions. Furthermore, identifying these determinants may aid in uncovering subtypes of miscarriage with distinct etiologies, supporting more targeted counselling and intervention strategies.

### Conclusion

This study highlights that predictive modelling for evidence-based patient management is feasible. Utilising a UK longitudinal dataset, we developed two models to predict a woman's pregnancy journey. To our knowledge, this is the first study to implement and combine these predictive approaches into a prediction of viable pregnancy long-term, and the first to integrate the resulting models into a pre-conception tool for holistic management of time to pregnancy, subfertility, and miscarriage for public use with evidenced-based advice. However, turning this tool into a widely applicable clinical tool, will require an extensive longitudinal perspective study and of course, extensive validation across a variety of medical conditions. Although our models have modest predictive power they provide a valuable guide to clinicians and patients. Since they are parametrised on real-world NHS data, they provide patients and clinicians with realistic expectations based on actual outcomes. In addition, they could provide a framework for evidence-based referral to fertility services. All in all, by tailoring referrals based on predictive insights and individual circumstances, this framework empowers healthcare providers to offer women the best possible support on their fertility journey.

## Contributors

SQ, AJD: study and questionnaire design, SQ, AJD, RSh, RSw: study execution, SQ, AJD, RSh, RSw, OK, SNLCK, CK, TNA: data curation, NJB: analysis design, JB, NJB: initial analysis and models, CK, RSh, RSw: data cleaning, CK, NJB: analysis and validation, CK, NJB, SQ: manuscript preparation, CK, SQ, NJB: critical editing and final draft, CK, SRh, OK, SQ, NJB: accessed and verified the data, CK, NJB, SQ: responsible for the decision to submit the manuscript.

## Data sharing statement

Original anonymised data is available upon request from SQ.

## Declaration of interests

The authors have no conflicts of interest to declare related to this study.
