# Supplementary material for: Fertility, time to pregnancy, and pregnancy outcomes among women with recurrent miscarriages in the UK: a prospective observational longitudinal study
Source: Lancet Reg Health Eur. 2025 Jun 26;55:101343. doi: 10.1016/j.lanepe.2025.101343 (PMC12268084; doi:10.1016/j.lanepe.2025.101343)
Supplement: Supplementary Materials [file mmc1.pdf]

Supplementary Text

Pregnancy Journey Analysis: fertility, time to pregnancy, and miscarriage from a UK follow up longitudinal study on women with recurrent pregnancy loss

Constandina Koki (ORCID: 0000-0002-3236-8876), Rebecca Shields, Rebecca Sweetman (ORCID: 0009-0005-8280-8939), James Boyle (ORCID: 0009-0004-6541-8712), Omar Khan (ORCID: 0000-0001-6851-3804), Sarah N Lim Choi Keung (ORCID: 0000-0001-9608-5990), Theodoros N Arvanitis (ORCID: 0000-0001-5473-135X), Adam J Devall (ORCID: 0000-0001-5632-079X), Nigel John Burroughs (ORCID 0000-0002-4632-1550), Siobhan Quenby (ORCID 0000-0003-3221-5471)

Contents

1 NHS referral process and data collection for this study **2**

1.1 NHS referral process . . . 2

1.2 Data collection . . . 2

2 Introduction: Model parametrisation **2**

2.1 Viable Pregnancy model . . . 2

2.1.1 Interpretation . . . 3

2.2 Time to pregnancy and Fertility model . . . 3

2.2.1 The model . . . 3

2.2.2 Likelihood and posterior distributions derivations . . . 4

2.2.3 Calculating the probability of being in the fertility problem group given that no pregnancy has occurred with time t_0 . . . 5

3 Viable pregnancy journey model: A Markov Chain model **5**

3.1 Probability of a viable pregnancy and number miscarriages . . . 7

4 Validation measures **7**

5 Model Calibration Statistics **8**

6 Supplementary Figures **9**

7 Supplementary Tables **14**

References **16**

1 NHS referral process and data collection for this study

1.1 NHS referral process

In the NHS (National Health Service, UK), patients who experience miscarriage initially present to their general practitioners (GPs) in primary care. The GPs act as healthcare purchasers, and secondary care in hospitals act as providers. GPs refer patients to secondary care services as needed, and within secondary care, some highly specialised services are designated as tertiary care—recurrent miscarriage clinics fall into this category.

Referral to a specialist recurrent miscarriage clinic generally follows specific criteria: a desire to conceive again, a history of two or three consecutive pregnancy losses, or a single second-trimester loss. In practice, some individuals are referred after just one early loss, particularly when there is significant emotional distress or a complex obstetric history. GPs also assess women for conditions such as polycystic ovary syndrome (PCOS), following NICE guidelines through clinical history, pelvic ultrasound, and hormone testing, National Institute for Health and Care Excellence (2025).

If a woman fails to conceive after 12 months of unprotected intercourse, she is referred to secondary care subfertility services. Women with symptoms suggestive of endometriosis or sexually transmitted infections are referred to gynaecology services for diagnosis and management. However, if women do not conceive while under the care of a recurrent miscarriage clinic, they must return to their GP to be referred to secondary care subfertility services. Direct referral between the miscarriage and subfertility services is not currently permitted under NHS pathways.

1.2 Data collection

In this study we analysed data from Recurrent Miscarriage clinics in University Hospital Coventry and Warwickshire (UHCW) (CV) and from clinics at Birmingham Women's Hospital Foundation Trust (BWH) and Imperial College Healthcare NHS Trust (Imperial) London (BL). The data was collected from May 2017 until November 2021. The prospective data collections were pre-registered[1]. 90% of clinic attendees consented to data collection. The study design, covariate list, questionaires, follow-up protocols and data collection methodology are discussed in Shields et al. (2022).

All had been referred with the goal of achieving a live birth. Because the NHS does not allow direct referral from recurrent miscarriage to subfertility services, we were able to observe natural conception events during the interim referral period. A high percentage (82%) of women entering our clinics had follow-up clinic visits, answered follow-up texts and/or telephone calls with information about further conceptions or lack of them. This provided important insights into natural conception delays and emerging subfertility.

2 Introduction: Model parametrisation

In the following sections, we provide details of the models and the Bayesian parameter inference methodology. We parametrised the viable pregnancy model and the time to pregnancy model on patients with specified covariates. Our dataset comprised 10 covariates (after removing covariates with a high number of missing data), specifically maternal age, maternal body mass index (BMI), previous number of miscarriages, number of previous live births, smoking (YES/NO), polycystic ovaries (PCOS, YES/NO), alcohol consumption (YES/NO), fibroids diagnosis (YES/NO), folic acid supplement intake (YES/NO) and ethnicity. Missing values for the training dataset were Missing Completely At Random (MCAR) as determined by the non-parametric test described in Jamshidian and Jalal (2010), implemented in the "MissMech" package in R, (Jamshidian et al., 2014).

2.1 Viable Pregnancy model

Let random variable $Y \in \{0, 1\}$ denote the end result of a pregnancy: Y is an indicator variable where $y_i = 1$ denotes a viable pregnancy (i.e., live birth, still birth and ongoing pregnancy with more than 24 weeks

[1] ISRCTN17732518; https://doi.org/10.1186/ISRCTN17732518. Ethics: REC Ref: 17/WM/0050: 17/WM/208

gestation) and $y_i = 0$ otherwise (i.e., a non-viable pregnancy or miscarriage), for patient i. Lower case of a random variable denotes an observed value. We use covariates X to explain/predict the probability of having a viable pregnancy. From an initial set of 10 covariates, we removed non-significant covariates (trained on the training data set), using a backward stepwise selection method, *i.e.,* removing the least significant covariate one after another, until we were left with only statistically significant variables at 5% significance level. After performing the backward selection, there are 5 covariates: “Age over 35 at conception time", *i.e.,* for women under the age of 35 the covariate is set to 0, “Polycystic Ovaries Syndrome", “BMI", “Previous Miscarriages", “Previous Live Births". The nonlinear dependence on maternal age is the simplest model to capture the increasing risk of aneuploidy related miscarriage, Magnus et al. (2019); de La Rochebrochard and Thonneau (2002).

The probability of a viable pregnancy is modelled as a logistic regression dependence on the covariates,

$$p\left(Y_i = 1\right) = \frac{\exp\left(x^i \beta\right)}{1 + \exp\left(x^i \beta\right)} \Leftrightarrow logit\left(p\left(Y_i = 1\right)\right) = x^i \beta,$$

where β are the parameters to be estimated, $\beta \in \mathbb{R}^{k \times 1}$, X is the design matrix, $X \in \mathbb{R}^{n \times k}$ and $x^i \in \mathbb{R}^{1 \times k}$ are the corresponding covariates values of the k covariates for the i-th patient.

We inferred the parameters using the package “stats" in R.

2.1.1 Interpretation

A positive coefficient (β_j, covariate j) of a covariate predictor means that the odds of the having a viable pregnancy increase as that covariate increases, a negative coefficient the odds decrease. The odds ratio (OR) is the exponentiated version of the coefficient, which indicates how the odds of the having a viable pregnancy change (relatively) under a one-unit increase in the predictor variable. In particular,

- Odds ratio greater than 1: the predictor increases the odds of having a viable pregnancy.
- Odds ratio less than 1: the predictor decreases the odds of of having a viable pregnancy.
- Odds ratio equal to 1: the predictor has no effect on the odds of having a viable pregnancy. This is an insignificant predictor.

In our case, as shown in Table 1 of the main text, the coefficient of the "Age (above 35)" is -0.11 and hence the odds ratio is $\exp -0.11 = 0.89$. Intuitively, after one year, the likelihood of having a viable pregnancy will decrease by 11% (1-0.89 = 0.11). On the contrary, the coefficient of "Previous Live births" is 0.19 giving an odds ratio 1.21 which in turn implies that having one previous live birth increases the likelihood of having a viable pregnancy by 21%.

2.2 Time to pregnancy and Fertility model

2.2.1 The model

The model belongs to the general class of models called "Cure Rate Models", Amico and Van Keilegom (2018) These models were initially developed to model survival times for diseases where a proportion of patients are "cured" and will never experience the event of interest, e.g., death (from the disease). In practice, the population is divided into two subgroups: the cured group, consisting of individuals who are cured and therefore expected to have a longer lifespan, and the susceptible group, which includes individuals, who are at risk and follow the survival distribution. A patients membership to a group is inferred from the data.

In our context, the 'cured group', or *fertility problem group*, consists of women (strictly the couple) identified as having fertility problems, who will never experience a pregnancy event regardless of how long they try to conceive; fertility issues may in fact stem from issues with either partner in the couple. The 'susceptible group', or *fertile group*, consists of women who will eventually experience a pregnancy event if they try for long enough. Let T be the time to pregnancy with cumulative distribution $F(t) = P(T \leq t)$ and probability density function $f(t)$. The general survival function for a pregnancy beyond time t is $S(t) := P(T > t) = 1 - F(t)$, and can be expressed as

$$S(t) = p + (1-p)\, S^*(t)$$

with p being the proportion of the "cured" individuals and $S^*(t)$ the survival function for the "non-cured" (susceptible) individuals. The cumulative distribution $F(t) = (1-p)(1 - S^*(t))$ is the product of the probability of being in the fertile group and having an event before t.

Since women may leave the study before they experience a pregnancy event or the study ends before a pregnancy event occurs, the data is right-censored. The general likelihood for these models takes the following form,

$$\mathcal{L}(\theta, p) = \prod_{i=1}^{N} \{(1-p)\, f^*(t)\}^{\delta_i} \{p + (1-p)\, S^*(t)\}^{1-\delta_i}$$

where the binary variable $\delta_i \in \{0, 1\}$ accounts for right-censored observations: $\delta_i = 0$ if no event is observed in patient i, and $\delta_i = 1$ if a pregnancy event is observed. Here $f^*(t) = -\frac{dS^*(t)}{dt}$ is the probability density function for the fertile group.

We assume that the time to pregnancy for women in the fertile group is exponentially distributed, with rate $\exp\left(\beta^T X_1\right)$, while we use a logistic regression model for patient classification into the fertility problem and fertile groups. The combined time to pregnancy and fertility model is given by,

$$T_i \sim \begin{cases} \text{Exp}\left(e^{\beta^T X_1^i}\right) & \text{with probability } \frac{1}{1+\exp\left(-\alpha^T X_2^i\right)} \quad \text{(fertile group)} \\ \infty & \text{with probability } \frac{\exp\left(-\alpha^T X_2^i\right)}{1+\exp\left(-\alpha^T X_2^i\right)} \quad \text{(fertility problem group)} \end{cases}$$

with $X_1, X_2 \subseteq X$ the covariates that explain/predict the time to pregnancy and the probability of having a fertility problem, respectively, $\beta \in \mathbb{R}^{k_1 \times 1}$ and $\alpha \in \mathbb{R}^{k_2 \times 1}$ the covariate coefficient vectors for the rate of the exponential distribution and the logistic regression coefficients.

The covariates set consisted of the “Age over 35 at the first consultation", *i.e.,* for women under the age of 35 the covariate is set to 0, “BMI over 25", *i.e.* for women with a BMI less than 25 the covariates is set to 0 , “Folic Acid", “Smoking" and “Previous Conceptions" as the sum of previous miscarriages and previous live births. We use simple nonlinear dependencies on age and BMI as indicated from previous studies, (Broughton and Moley, 2017; Ramlau-Hansen et al., 2007), specifically fertility decreases in late 30s and both low and high BMI impact fertility. We did not pursue model selection methods to refine these dependencies as the sample sizes are insufficient to warrant such an analysis.

2.2.2 Likelihood and posterior distributions derivations

The likelihood of the model (with right censored data) is

$$\begin{aligned} L(t, \delta | \alpha, \beta, x) = & \prod_{\text{pregnant}} \left(\frac{1}{1 + \exp(-\alpha^T x_2^i)} e^{\beta^T x_1^i} \exp(-e^{\beta^T x_1^i} t_i) \right) \\ & \prod_{\text{no event}} \left(\frac{\exp(-\alpha^T x_2^i)}{1 + \exp(-\alpha^T x_2^i)} + \frac{1}{1 + \exp(-\alpha^T x_2^i)} \exp(-e^{\beta^T x_1^i} t_i) \right) \\ = & \prod_{i=1}^{N} \left(\frac{1}{1 + \exp(-\alpha^T x_2^i)} e^{\beta^T x_1^i} \exp(-e^{\beta^T x_1^i} t_i) \right)^{\delta_i} \\ & \left(\frac{\exp(-\alpha^T x_2^i)}{1 + \exp(-\alpha^T x_2^i)} + \frac{1}{1 + \exp(-\alpha^T x_2^i)} \exp(-e^{\beta^T x_1^i} t_i) \right)^{1-\delta_i} \end{aligned}$$

We use a Bayesian methodology to infer the model parameter posteriors using a Markov chain Monte Carlo (MCMC) algorithm with Metropolis Hasting updates for all the parameters. The algorithm was implemented in PYTHON. We used multivariate normal priors on parameters α and β.

2.2.3 Calculating the probability of being in the fertility problem group given that no pregnancy has occurred with time t_0

A failure to have a pregnancy within time t_0 could be due to either the couple have a fertility problem, or, although fertile, insufficient time had elapsed. Let I denote the case that a couple belongs to the fertility problem group (the couple will not experience an event), *i.e.*, $T = \infty$ and F the case that couple belongs to the fertile group, *i.e.,* the time to event $T < \infty$. The probability of a couple being in the fertility problem group given that the time to pregnancy is greater than t_0 can be derived using the Bayes theorem,

$$
\begin{aligned}
P(I \mid T \geq t_0,\, X = x) &= \frac{P(T \geq t_0 \text{ and } I \mid X = x)}{P(T \geq t_0 \mid X = x)} \\
&= \frac{P(I \mid X = x)}{P(I \mid X = x) + P(T \geq t_0 \mid F, X = x)\, P(F \mid X = x)} \\
&= \frac{\frac{\exp\left(-\alpha^T X_2\right)}{1+\exp\left(-\alpha^T X_2\right)}}{\frac{1-F_{Exp}(t)}{1+\exp\left(-\alpha^T X_2\right)} + \frac{\exp\left(-\alpha^T X_2\right)}{1+\exp\left(-\alpha^T X_2\right)}} \\
&= \frac{\exp\left(-\alpha^T X_2\right)}{\exp\left(-\exp\left(\beta^T X_1\right) t\right) + \exp(-\alpha^T X_2)}
\end{aligned}
$$

This probability can be evaluated for a specific patient using the posterior mean estimations of α and β, or estimated using using Monte Carlo simulations.

3 Viable pregnancy journey model: A Markov Chain model

To determine if a couple will have a viable pregnancy within a period of time (with or without intervening miscarriages) we need to integrate the viable pregnancy model, 2.1 and the time to pregnancy/fertility model 2.2. We model time in terms of consecutive menstrual cycles, *i.e.* we use our models to calculate the probability of a pregnancy per cycle and the probability that that pregnancy is viable or ends in miscarriage. The model is general, so could be used for women who have not experienced a miscarriage if appropriately parametrised from a natural pregnancy dataset. We parametrise the model probabilities from our two validated predictive models for women with a history of miscarriage.

We use a fecundity model, *i.e.* modelling the probability of a pregnancy per menstrual cycle (fecundity), assuming a regular menstrual cycle of 28 days. Irregular cycles could be included if a distribution for inter cycle time is available, and dependencies on covariates such as PCOS inferred (our dataset did not incorporate menstrual cycle data). We discretise time into cycles of $\Delta = 28$ days, and the maternal age is advanced by 28 days per cycle, i.e. our model updates age and the associated probabilities of pregnancy, miscarriage and infertility each month. We then have a discrete time Markov chain (MC), using the survival function $\exp\left\{-\left(e^{\beta^T X}\,\Delta\right)\right\}$ for the probability of no pregnancy in that cycle. Since miscarriages typically occur within the first trimester, we disperse miscarriages over the first 3 months with equal weight. We ignore miscarriage in the 2nd trimester, thus a pregnancy of 3 months duration will transition to a viable pregnancy after the 2nd trimester, Diagram S1. The couple can be in any of 8 states given the number k of previous miscarriages, Diagram S1. Specifically,

- State N: Not pregnant and capable of becoming pregnant
- State I: Infertile, *i.e.* the couple have a fertility problem so pregnancy is not possible
- States P_s, $s = 1, 2, 3$: Pregnant in sth month of the first trimester.
- State P_B: Pregnant in second trimester.
- State V: Viable pregnancy (> 24 weeks) [2].

[2] In practice, state P_B and V are merged together, *i.e.* we do not model miscarriage events in the 2nd trimester as negligible compared to 1st trimester rates

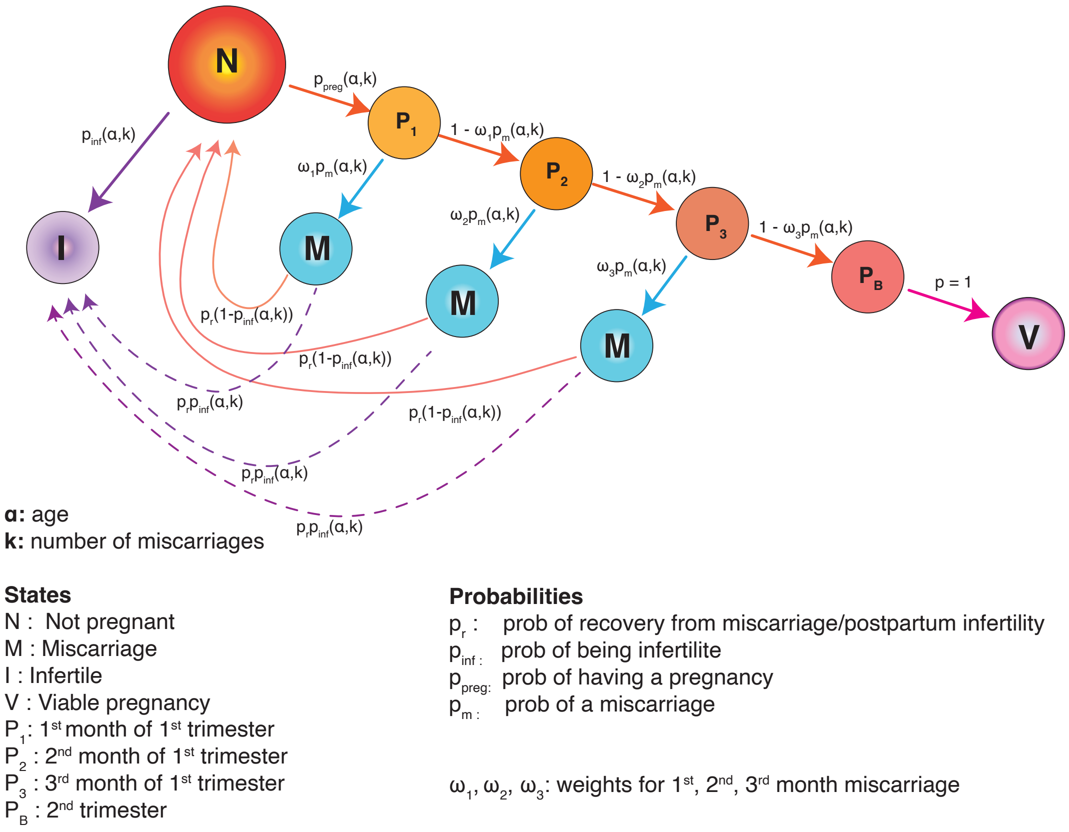

Diagram S1. Viable pregnancy journey. Schematic showing the states (circles) of the Markov chain model and the transitions and associated transition probabilities. At each cycle/month ($\Delta = 28$ days), the woman's age is increased by Δ, $a \to a + \Delta$. If a miscarriage occurs, the number of miscarriages is incremented by one, $k \to k + 1$. Pregnancy in the second trimester P_B is distinguished from viable pregnancy in the schematic, but pooled in the simulations since we ignore miscarriage in the 2nd trimester, so a 2nd trimester pregnancy would transition to a viable pregnancy after 3 months.

- State M: Miscarriage.

Let $P(a, S)$ be the probability of being in state S at age a, a increasing from the initial age a_0. The patient state $S = (S_1, k)$ comprises 2 variables: the pregnancy state $S_1 \in \{N, I, P_s, V, M\}$, and the number of previous miscarriages, $k = 0, 1, 2, ..m$, setting a maximum m, large enough so that it is never reached in practice. For women with RM, $k > 0$. The possible transitions from each state are (age a advances by Δ per time step) summarised below, with the relevant probabilities:

- $N \xrightarrow{p_{inf}} I$
- $N \xrightarrow{p_{preg}} P_1$
- $P_1 \xrightarrow{1-\omega_1 p_m} P_2$
- $P_1 \xrightarrow{\omega_1 p_m} M$ and $k \to k+1$
- $P_2 \xrightarrow{1-\omega_2 p_m} P_3$
- $P_2 \xrightarrow{\omega_2 p_m} M$ and $k \to k+1$
- $P_3 \xrightarrow{1-\omega_3 p_m} P_B$
- $P_3 \xrightarrow{\omega_3 p_m} M$ and $k \to k+1$
- $P_B \xrightarrow{p=1} V$
- $M \xrightarrow{p_r(1-p_{inf})} N$
- $M \xrightarrow{p_r p_{inf}} I$

Note that the infertile and viable pregnancy states are absorbing, *i.e.* once a woman enters either of

these states, she remains there and cannot transition to any other state. The transition probability from the miscarriage state incorporates a delay (mean Δ/p_r) to model the time after a miscarriage event before the couple are either trying to get pregnant or the woman is capable of getting pregnant.

Let $X_{(a,k)}$ be the cofactor vector (categorical data mapped to binary) with age a, miscarriages k. The transition probabilities are given by:

- **Probability infertility transition/cycle**. $p_{inf}(a,k) = \frac{1}{1+\exp(\alpha^T X_{(a+\Delta,k)})} - \frac{1}{1+\exp(\alpha^T X_{(a,k)})}$, the difference in the infertility probability over a (28 day) cycle. Transitions occur from both N and M states.

- **Pregnancy probability per cycle**. $p_{preg}(a,k) = 1 - \exp\left\{-\left(e^{\beta^T X_{(a,k)}}\,\Delta\right)\right\}$.

- **Probability miscarriage occurs.** $p_M(a,k) = \frac{\exp(\gamma^T X_{a,k})}{1+\exp(\gamma^T X_{a,k})}$, the weights ω_s disperse the miscarriage over the first trimester, $\sum_{s=1}^{3}\omega_s = 1$; thus the average time to miscarriage is $\Delta\sum_{s=1}^{3} s\omega_k$.

- **Transition probability to viable pregnancy**. The transition probability from P_3 to viable pregnancy V (through the 2nd trimester) is $(1-\omega_3 p_M(a,s))$. Since the age changes the probability of a viable pregnancy, the probability that a new pregnancy at age a will successfully reach the 2nd trimester is,

$$(1-\omega_1 p_M(a,k))\,(1-\omega_2 p_M(a+\Delta,k))\,(1-\omega_3 p_M(a+2\Delta,k))\,.$$

  A MC with viable pregnancy probability $(1-p_M(a,k))$, thus exactly reproducing the inferred miscarriage model, could be constructed, but the differences are negligible.

- **Recovery from miscarriage/postpartum subfertility**. We assume a constant recovery probability per cycle after a miscarriage, and use $p_r = 1/2$, so the average postpartum subfertility period is 2 months (geometrically distributed recovery time, with mean number months for recovery = $p_r/(1-p_r)^2$). We have no data on age dependency or other covariates, so assume a constant probability.

3.1 Probability of a viable pregnancy and number miscarriages

We are interested in computing the probability of a woman of age a achieving a viable pregnancy, *i.e.* the probability of being in state V at some later time (corresponding to a step in the MC). Three factors affect this probability, all of which deteriorate with age, the probability of being fertile, the probability of a pregnancy at the next cycle, and the probability of a viable pregnancy per pregnancy. Define the probability of a viable pregnancy by age a_* of a woman currently aged a_0, with previous miscarriages k_0, (suppressing other covariates)

$$P_V(a_*;a_0,k_0) = \sum_{k=k_0}^{m}\sum_{a=a_0}^{a_*} P(a,(V,k))$$

and the distribution of (additional) miscarriages before a viable pregnancy,

$$P_{seq.M}(a_*,M=k|V;a_0,k_0) = \frac{1}{P_V(a_0,k_0)}\sum_{a=a_0}^{a_*} P(a,(V,k))$$

4 Validation measures

Model performance was calibrated using the receiver operating characteristic (ROC) analysis. We quote area under the curve (AUC) which measures model performance across all choices of threshold (for instance on sensitivity or specificity). An AUC of 1 would be perfect prediction, an AUC of 0.5 would be purely random predictions and an AUC>0.5 indicates that the prediction is better than random.

To validate the model we used three different methods:

1. Leave-One-Out (LOO) method: We split our data (CV dataset only) into a training set and a test set. Then, we trained our models using the training set and used the test set to measure the predictive performance of the inferred model. In LOO, the training set consists of all observations but one, thus,

the size of the training set is $N - 1$. We repeated this method N times to obtain predictions for all observations. The AUC on pooled LOO predictions then measures the predictive performance of our model.

2. Leave-k-Out (LkO) method: Instead of leaving 1 observation out of the training set each time and evaluate the predictive performance of our model, we split our data set into $k = 10$ folds/partitions and use the $k - 1 = 9$ folds as a training set and the other one as a test set. Each fold is used once as a validation, while the remaining $k - 1 = 9$ folds form the training set. The LkO AUC is the average of the 10 AUC values for the 10 folds; Figure 3e in the main text, uses a test set of size n = 66 from the CV dataset.

3. External validation: We trained the model using the whole CV dataset and validated on the external BL dataset (test set), using again the AUC measure.

The ROC curves and AUCs (including the 95% confidence intervals) were performed with the "pROC" package in R for the " Viable Pregnancy model", and with a bespoke bootstrapping methodology for the "Time to pregnancy and Fertility" model (implemented in PYTHON).

5 Model Calibration Statistics

We follow Van Calster et al. (2019), and references therein, to assess the calibration performance of our two predictive models. We report the following values.

1. Calibration-in-the-large (or mean calibration): This is the average predicted risk compared to the overall event rate observed in the test set. The calibration intercept, which is an assessment of calibration-in-the-large, has a target value of 0; negative values suggest overestimation, whereas positive values suggest underestimation. When the average predicted risk is higher than the overall event rate (calibration-in-the-large > 0), the predictive model overestimates risk in general. In contrast, underestimation occurs when the observed event rate is higher than the average predicted risk (calibration-in-the-large < 0).

2. Calibration Slope: The calibration slope evaluates the spread of the estimated risks, or the uniformity of predicted risk across level of risk. It has a target value of 1. A slope < 1suggests that estimated risks are too extreme, i.e., too high for patients who are at high risk and too low for patients who are at low risk. A slope > 1 suggests the opposite, *i.e.* that risk estimates are too moderate.

The calibration measures for our models are reported in Tables 2 and 3 of the main text, and Supplementary Tables S1, S2 of the additional models. The calibration values for the two models are not significantly different than the target values, so there is no evidence of overfitting or over estimation. Thus, there is insufficient evidence to warrant a model re-calibration. Furthermore, given the constraints of our sample sizes introduce large noise/wide confidence intervals a re-calibration would have introduced more noice and hence we do not proceed any further with this analysis.

All calibration measures were calculated using the "CalibrationCurves", (Van Calster et al., 2016; De Cock et al., 2023) and "rms" packages in R.

6 Supplementary Figures

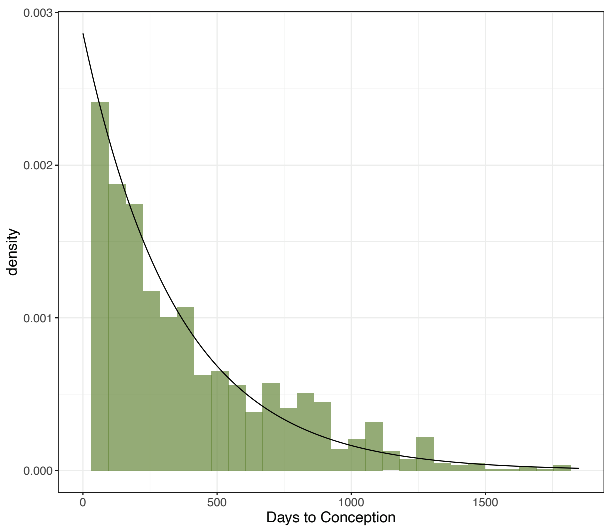

Figure S1: Days to Conception (histogram and exponential density plot) Mean = 348, sd = 339.

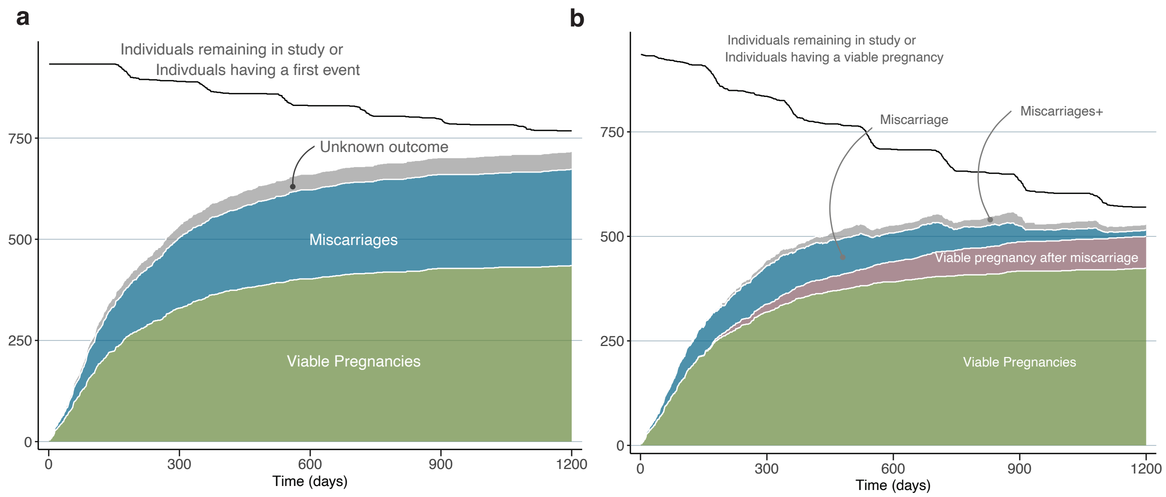

Figure S2: Cumulative outcomes for the training set only (CV dataset): (a) Stacked first event outcome with time on programme (counts). Cumulative events only in CV dataset showing (stacked) viable pregnancy (>24 weeks) (green), a miscarriage (blue) and an ongoing or a reported pregnancy but without confirmed outcome (grey). (b) Decomposition of couples that have a viable pregnancy as first event on study (green) or after a miscarriage on study (pink). The number of couples with one miscarriage (blue) or multiple miscarriages (grey) on study before having a viable pregnancy is shown through by time.

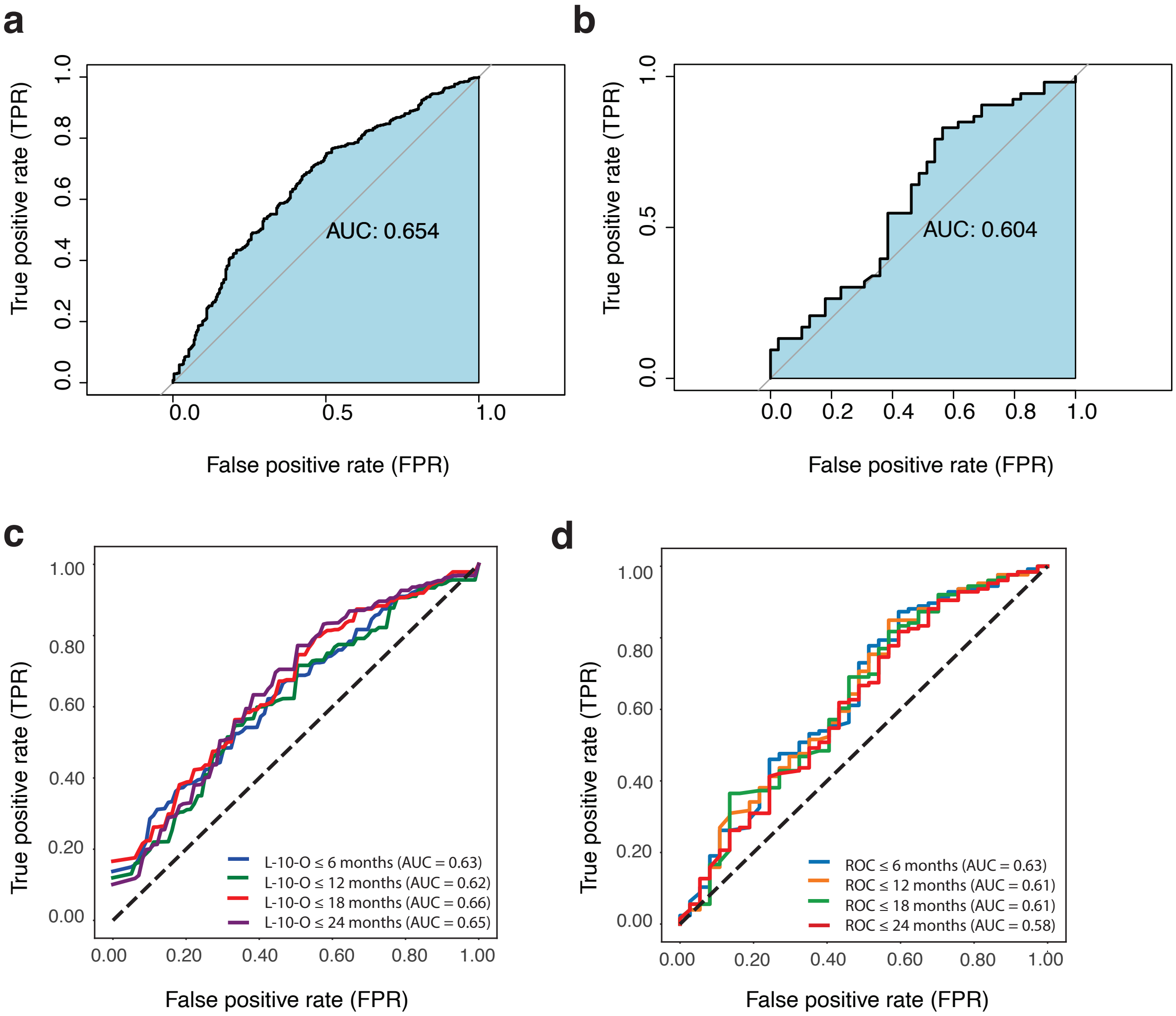

Figure S3: Assessing viable pregnancy model's (a,) and time to pregnancy and fertility model's (c,d) predictive/discrimination performance using the covariates "Age", "BMI", "PCOS", "HisMiscarriages", "HisLiveBirths", "Folic Acid", and "Smoking", with Leave-One-Out (a), Leave-10-Out (c) and external validation with BL dataset (b, d) methodology. The predictive performance is almost identical to the models with only the statistically significant covariates.

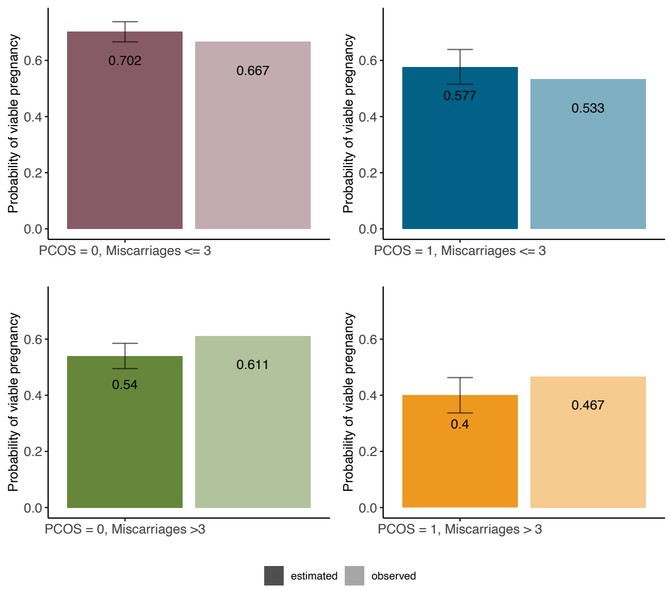

Figure S4: Assessing viable pregnancy model performance on patient subgroups by 2 covariates: PCOS and previous miscarriages. For instance, the probability of a viable pregnancy for women with PCOS and a history of at least 3 miscarriages versus women with no PCOS and a history of less than 3 miscarriages is reduced by 42.9. Trained on CV:RFE002 and validated on CV:RFE003+BL:RFE005. Dark columns show the averaged predicted probabilities of a viable pregnancy for each subgroup of BL dataset. Error bars denote the standard deviation of the predicted probabilities.

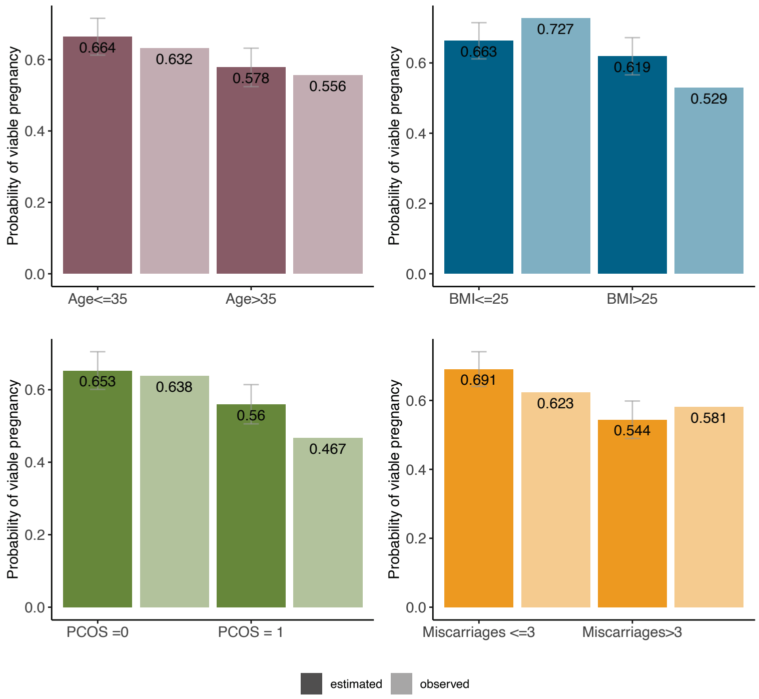

Figure S5: Accessing model performance on patient subgroups. Trained on CV and validated on BL. Model estimations are derived when trained with CV data set. Dark columns show the averaged predicted probabilities of a viable pregnancy for each subgroup of BI dataset. Error bars denote the standard deviation of the predicted probabilities.

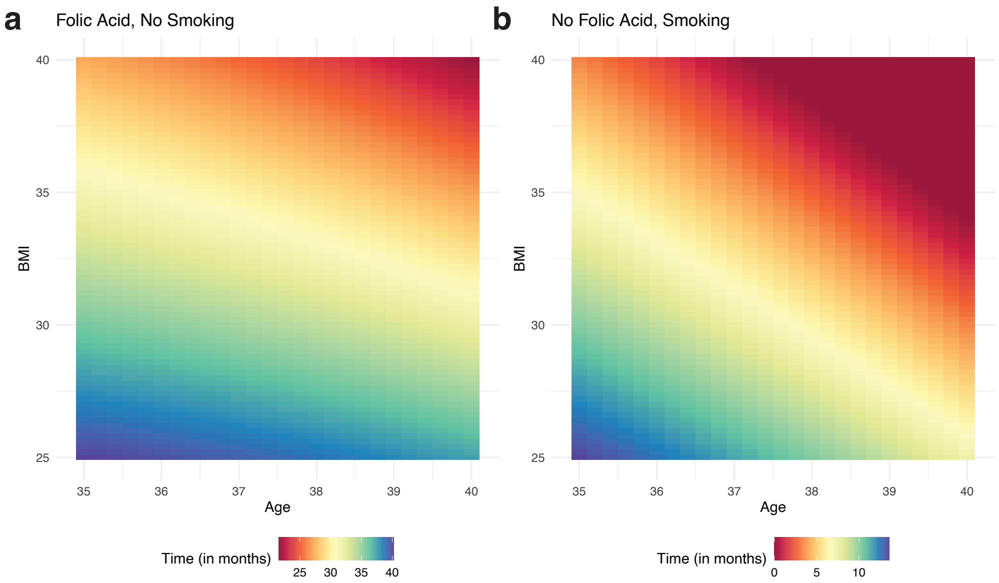

Figure S6: Time (in months) for a couple without a conception to reach the referral threshold of 70% for the predicted probability of having a fertility problem against age and BMI. (a) Women taking folic acid supplements, aren't smoking. (b) Women who are not taking folic acid supplements and smoke. In both cases women have a history of 2 previous conceptions. Note the difference in time scale in panels.

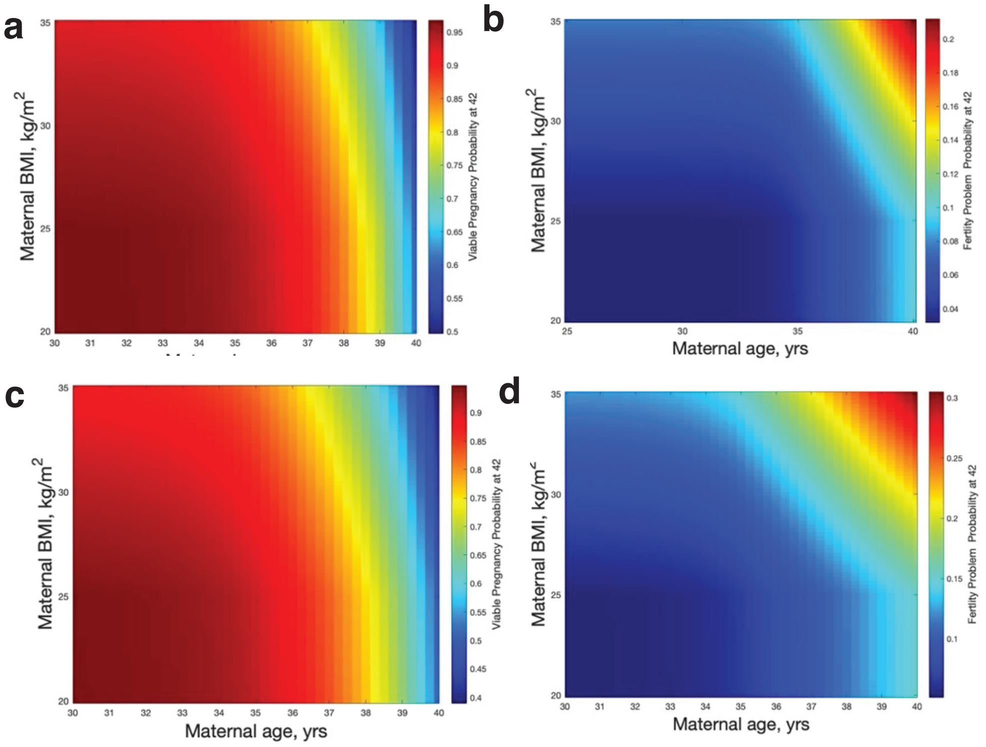

Figure S7: Combined model prediction for viable pregnancy by the age of 40 with BMI and current age. (a,c) Probability of a viable pregnancy by age 40. (b,d) Probability of having a fertility problem by age 40. Other covariates are: (a,b) non-smoker, taking folic acid supplements, no history of PCOS, 2 previous miscarriages and no previous live births. (c,d) non-smoker, not taking folic acid supplements, history of PCOS, 4 previous miscarriages and no previous live births.

7 Supplementary Tables

Covariate	Mean	Standard Error	p-value
Intercept	2.45	0.49	$< 0 \cdot 0001$
Age	-0.13	0.04	$< 0 \cdot 0001$
BMI	-0.03	0.02	0.050
PCOS	-0.65	0.25	0.012
Miscarriage History	-0.23	0.05	0.0013
Live Births History	0.26	0.13	0.052
	**Area Under Curve (95% CI)**		
LOO	0.65 (0.60, 0.69)		
BL	0.64 (0.50, 078)		
	**Calibration (95% CI)**		
Calibration-in-the-large	-0.49 (-0.98, 0.00)		
Calibration slope	0.63 (0.50, 0.76)		
	**Observed/Expected Ratio (95% CI)**		
BL	0.84 (0.70, 1.00)		

Table S1: Viable pregnancy model for analyzing first event pregnancies when trained on CV dataset and validated with Leave-one-out (LOO) and externally on BL dataset. Averaged predicted risk (calibration in the large), calibration intercept and observed versus expected (predicted) ratio, are reported for external validation (95% CI).

Time to Pregnancy			
**Covariate**	**Mean (90% BCI)**		**Tail Probabilities**
Intercept	-5.27 (-5.46 -5.07)		0.00
Age	-0.04 (-0.09, -0.0031)		0.042
BMI	-0.01 (-0.04, 0.023)		0.37
Folic Acid	-0.21 (-0.39, -0.043)		0.025
Smoking	0.26 (-0.14, 0.63)		0.86
PCOS	-0.10 (-0.37, 0.16)		0.28
Miscarriage History	-0.07 (-0.11, -0.022)		0.0051
Live Births	-0.03 (-0.13, 0.081)		0.33
**Fertility Problem**			
**Covariate**	**Mean (90% BCI)**	**Odds Ratio**	**Tail Probabilities**
Intercept	1.69 (0.93, 2.46)	5.43	1.000
Age	-0.17 (-0.31, 0.01)	0.85	0.057
BMI	-0.09 (-0.17, 0.013)	0.91	0.068
Folic Acid	0.96 (0.26, 1.718)	2.60	0.99
Smoking	-1.71 (-2.49, -0.91)	0.18	0.0013
PCOS	-0.17 (-0.96, 0.76)	0.84	0.34
Miscarriage History	0.34 (0.08, 0.65)	1.41	0.99
Live Births	0.12 (-0.32, 0.61)	1.12	0.64
**Area Under Curve (95% CI)**			
	**LkO**	**External BI**	
6 months	0.63 (0.55, 0.66)	0.63 (0.53, 0.71)	
12 months	0.62 (0.56, 0.67)	0.61 (0.52, 0.70)	
18 months	0.66 (0.58, 0.69)	0.61(0.50, 0.69)	
24 months	0.65 (0.57, 0.68)	0.58 (0.49, 0.68)	
**Calibration Statistics (95% CI)**			
Calibration-in-the-large	-0.34 (-0.69, 0.00)		
Calibration slope	1.14 (0.07, 2.21)		

Table S2: Time to pregnancy and fertility model inferred with all covariates. We report the posterior mean estimations for each covariate (90% Bayesian Credible Interval) and the associated posterior tail probabilities, analogous to p-values. Model was trained on the CV dataset and validated on the BL dataset or through Leave-k-Out (LkO, k = 10) for achieving a pregnancy within a specified interval, and we report the Area Under Curve (95% CI). Averaged predicted risk (calibration in the large), calibration intercept and observed versus expected (predicted) ratio, are reported for predicting a pregnancy within 12 months using external validation. Averaged predicted risk (calibration in the large), calibration intercept and observed versus expected (predicted) ratio, are reported for predicting a pregnancy within 12 months using external validation.

References

Amico, M. and I. Van Keilegom (2018). Cure models in survival analysis. *Annual Review of Statistics and Its Application 5*, 311–342.

Broughton, D. E. and K. H. Moley (2017). Obesity and female infertility: potential mediators of obesity's impact. *Fertility and Sterility 107*(4), 840–847.

De Cock, B., D. Nieboer, B. Van Calster, E. Steyerberg, and Y. Vergouwe (2023). The calibrationcurves package: assessing the agreement between observed outcomes and predictions. `https://cran.r-project.org/package=CalibrationCurves`. R package.

de La Rochebrochard, E. and P. Thonneau (2002, 06). Paternal age and maternal age are risk factors for miscarriage; results of a multicentre european study. *Human Reproduction 17*(6), 1649–1656.

Jamshidian, M. and S. Jalal (2010). Tests of homoscedasticity, normality, and missing at random for incomplete multivariate data. *Psychometrika 75*, 649–674.

Jamshidian, M., S. Jalal, and C. Jansen (2014). Missmech: An r package for testing homoscedasticity, multivariate normality, and missing completely at random (mcar). *Journal of Statistical Software 56*(6), 1–31.

Magnus, M. C., A. J. Wilcox, N.-H. Morken, C. R. Weinberg, and S. E. Håberg (2019). Role of maternal age and pregnancy history in risk of miscarriage: prospective register based study. *BMJ 364*.

National Institute for Health and Care Excellence (2025). Polycystic ovary syndrome. `https://cks.nice.org.uk/topics/polycystic-ovary-syndrome`. Accessed: 2025-05-01.

Ramlau-Hansen, C., A. Thulstrup, E. Nohr, J. Bonde, T. Sørensen, and J. Olsen (2007). Subfecundity in overweight and obese couples. *Human Reproduction 22*(6), 1634–1637.

Shields, R., O. Khan, S. Lim Choi Keung, A. J. Hawkes, A. Barry, A. J. Devall, S. D. Quinn, S. D. Keay, T. N. Arvanitis, D. Bick, and S. Quenby (2022). Quantitative assessment of pregnancy outcome following recurrent miscarriage clinic care: a prospective cohort study. *BMJ Open 12*(2).

Van Calster, B., D. J. McLernon, M. van Smeden, L. Wynants, E. W. Steyerberg, P. Bossuyt, G. S. Collins, P. Macaskill, K. G. M. Moons, and A. J. Vickers (2019). Calibration: the achilles heel of predictive analytics. *BMC Medicine 17*(1), 230.

Van Calster, B., D. Nieboer, Y. Vergouwe, B. De Cock, M. Pencina, and E. Steyerberg (2016). A calibration hierarchy for risk models was defined: from utopia to empirical data. *Journal of Clinical Epidemiology 74*, 167–176.
